# Impact of timing of adjuvant chemotherapy on survival for early-stage node-negative small-cell lung cancer

**DOI:** 10.1016/j.xjon.2025.07.013

**Published:** 2025-07-24

**Authors:** Arian Mansur, Adele J. Lee, Alexandra L. Potter, Jacob M. Sands, Catherine B. Meador, Michael Lanuti, Chi-Fu Jeffrey Yang

**Affiliations:** a Division of Thoracic Surgery, Department of Surgery, Mass General Brigham, Boston, Mass; b Department of Medical Oncology, Dana-Farber Cancer Institute, Boston, Mass; c Mass General Brigham Cancer Institute, Boston, Mass

**Keywords:** adjuvant chemotherapy, small cell lung cancer, optimal timing

## Abstract

**Background:**

The relationship between timing of adjuvant chemotherapy and survival for early-stage, node-negative small cell lung cancer is not well defined, and no formal guidelines exist. We sought to evaluate whether increasing the time between surgery and adjuvant chemotherapy for pathologic stage I-IIA SCLC would be associated with worse survival.

**Methods:**

The association between timing of adjuvant chemotherapy and survival for patients with pathologic stage I-IIA (pT1-2N0M0) SCLC who have 1 or fewer co-morbidities in the National Cancer Database from 2004-2021 was assessed using multivariable Cox regression analysis with penalized smoothing spline functions and propensity score-matched analysis. Adjuvant chemotherapy received within 21-40 days of surgery was classified as “earlier” while adjuvant chemotherapy received 41-90 days after surgery was classified as “later.”

**Results:**

Of 927 patients who met study criteria, the median time to adjuvant chemotherapy was 41 days (interquartile range, 34, 53). In multivariable and propensity score-matched analyses, there was no significant difference in overall survival between earlier and later adjuvant chemotherapy. These findings were consistent when limited to patients who were discharged within 4 days of surgery or when adjusting for minimally invasive surgical approaches.

**Conclusions:**

In this national analysis of patients with early-stage node-negative SCLC, there was no significant difference in overall survival based on the timing of adjuvant chemotherapy.

**Figure:**
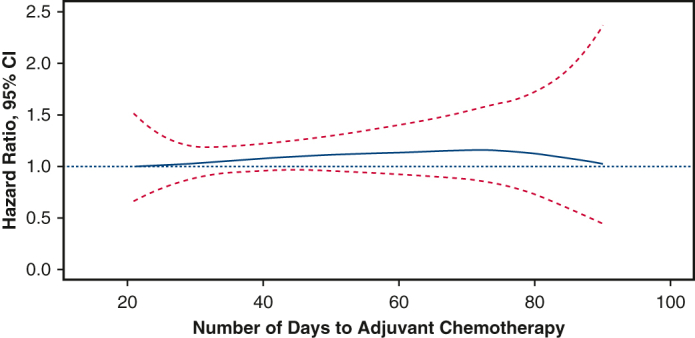
Smoothing spline for time elapse from surgery to adjuvant chemotherapy for pathologic T1-2N0M0 small cell lung cancer.

**Central Message**

In this national study, there was no association between the timing of adjuvant chemotherapy and overall survival in patients with pathologic T1-2N0M0 small cell lung cancer.

**Perspective**

Current guidelines recommend that patients with early-stage node-negative small cell lung cancer (SCLC) receive surgery followed by adjuvant chemotherapy, but there is no guidance on the timing of chemotherapy initiation. In this National Cancer Database study of well-selected patients with pathologic T1-2N0M0 SCLC, no significant association was observed between chemotherapy timing and overall survival.

Current guidelines recommend that patients with early-stage node-negative small cell lung cancer (SCLC) should receive surgery followed by adjuvant chemotherapy.^1^, ^2^, ^3^, ^4^ However, the optimal timing of adjuvant chemotherapy after surgery remains unknown. To our knowledge, there have been no studies, prospective or retrospective, evaluating the optimal timing of adjuvant chemotherapy for pathologic T1-2N0M0 SCLC. While patients typically receive adjuvant chemotherapy around 6-9 weeks after surgery for non–small cell lung cancer (NSCLC),^5^ SCLC is more aggressive than NSCLC, and patients may benefit from earlier initiation of adjuvant treatment.^6^

Given the absence of data, we sought to assess the association between the timing of adjuvant chemotherapy and overall survival in patients with pT1-2N0M0 SCLC. We hypothesized that minimizing the time between surgery and initiation of adjuvant chemotherapy would be associated with increased survival when compared to later adjuvant chemotherapy for pT1-2N0M0 SCLC patients.

## Methods

### National Cancer Database

The National Cancer Database (NCDB) is a joint project of the Commission on Cancer (CoC) of the American College of Surgeons and the American Cancer Society. The NCDB collects data from hospital registries at over 1500 CoC accredited facilities nationwide. The data captured from the NCDB are estimated to include over 80% of newly diagnosed lung cancer cases nationwide^7^ and constitutes more than 24 million patient records.^8^ Clinical staging information is recorded in the NCDB using the sixth, seventh, and eighth edition of the American Joint Committee on Cancer TNM classifications for the years of study inclusion.^9^ For this study, staging was reclassified using best available data according to the American Joint Committee on Cancer eighth edition.^10^

### Study Design

This retrospective analysis was approved by the Institutional Review Board of Mass General Brigham (No. 2020P004110, approval date: 2/2/2021). From a deidentified 2022 NCDB participant user file, all patients in the NCDB diagnosed with pathologic T1-2N0M0 SCLC from 2004 to 2021 were identified using International Classification of Diseases for Oncology, 3rd edition histology and topography codes. The NCDB does not have data on whether the diagnosis of SCLC was made prior to surgical resection (eg by CT-guided biopsy prior to surgery); however, all patients included in this study had a diagnosis of SCLC as confirmed by pathologic evaluation following surgical resection. The study period was chosen because we had data on the Charlson/Deyo comorbidity condition (CDCC) score only for cases diagnosed in 2004 onwards, and survival data were available for patients diagnosed up to 2021 at the time of analysis.

Methods of follow-up have been described previously (eg, reports from physician follow-up, program inpatient or outpatient services, death certificates).^11^ To minimize confounding, the cohort was limited to patients who were initially diagnosed with a single malignancy of SCLC. Patients who died within 6 months of surgery, were readmitted within 30 days of discharge, or those who had more than one comorbidity were excluded from analysis to minimize selection bias. Patients who were not diagnosed or treated at the reporting facility were excluded because the CoC does not require follow-up for these patients. Additional exclusion criteria included incomplete resection, less than three lymph nodes evaluated, missing data on facility type, patients who received induction therapy, adjuvant immunotherapy, or adjuvant chemotherapy within three weeks of surgery, and patients who were treated with palliative intent. We excluded patients who received immunotherapy (induction or adjuvant) from analysis as this was not standard of care during the study period and any patient receiving immunotherapy would likely be on a clinical trial and may have different baseline characteristics.

The primary outcome was overall survival (OS), which was defined from time of surgery to last follow-up or death.

**Figure E1:**
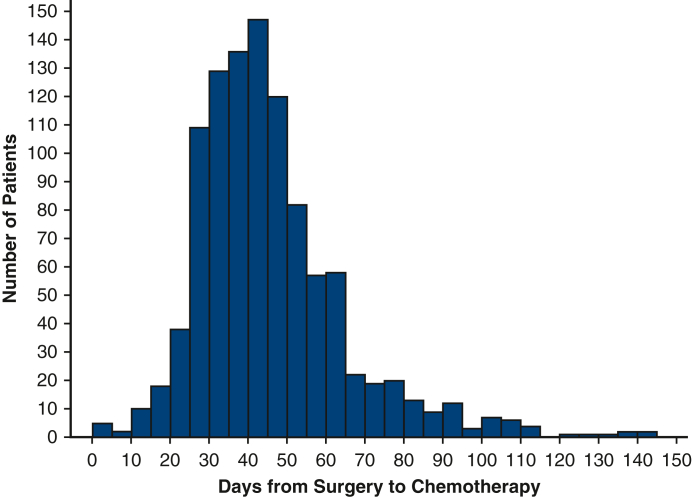
Distribution of time from surgery to initiation of adjuvant chemotherapy among patients with early-stage, node-negative small cell lung cancer in our study cohort.

Adjuvant chemotherapy was defined as chemotherapy administered between 3 weeks (21 days) and 3 months (90 days) following surgery. Twenty-one days (eg, 3 weeks) was used as the lower cutoff to account for the length of typical post-surgical recovery and to reduce selection bias (eg patients receiving adjuvant chemotherapy less than 21 days from surgery may have a performance status far exceeding the national average). Ninety days was chosen as the upper threshold after evaluating the distribution of time to adjuvant chemotherapy for the entire cohort (Figure E1). These bounds also take into consideration the real-world, nonclinical trial setting where patients may experience delays between surgery and initiation of adjuvant chemotherapy as a result of postoperative recovery from complications, delays in referral and/or consultation, and patient preference.^12^

### Statistical Analysis

The number of days from surgery to adjuvant chemotherapy was identified for each patient. Evaluation of the impact of timing of adjuvant chemotherapy was performed by first categorizing patients into “earlier” (21-40 days from time of surgery to adjuvant chemotherapy) and “later” (41+ days). Forty days was chosen as the upper cut off because the distribution of timing of adjuvant chemotherapy for the entire cohort showed a median time to adjuvant chemotherapy of 41 days. Baseline characteristics between these two groups were compared using Student's *t* test or Wilcoxon rank sum test for continuous variables and Pearson's χ^2^ test or Fisher's exact test for categorical variables.

A logistic regression model that included age, sex, race, year of diagnosis, insurance type, facility type, distance from facility, CDCC score, tumor size, tumor location, surgery type, pathologic T stage, adjuvant radiation, and facility location was used identify predictors of later adjuvant chemotherapy. Adjuvant radiation was stratified by prophylactic cranial irradiation alone, radiation to the lung alone, or both prophylactic cranial irradiation and radiation to the lung.

Differences in OS between earlier and later adjuvant chemotherapy groups were assessed with the Kaplan-Meier method and the log-rank test. Next, we modeled time from surgery to adjuvant chemotherapy with penalized smoothing splines with three degrees of freedom. Penalized smoothing splines have the advantage of flexibility and can capture potential nonlinearities in the dose-response between time from surgery to adjuvant chemotherapy and mortality rates.^13^^,^^14^ We used fitted models to plot the hazard ratio as a function of days from surgery to adjuvant chemotherapy with 21 days from surgery to adjuvant chemotherapy as the reference. We controlled for a priori specified covariates that could plausibly confound the association between time from surgery to adjuvant chemotherapy and mortality. These included: age, sex, race, year of diagnosis, insurance type, facility type, distance from hospital, CDCC score, tumor size, pathologic T stage, tumor location, surgery type, and adjuvant radiation. Subsequently, a multivariable Cox proportional hazards model was used to evaluate differences in overall survival between the earlier and later adjuvant chemotherapy groups, adjusting for the same above-mentioned covariates. In a secondary analysis, chemotherapy timing was modeled as a categorical variable using 2-week intervals (eg, 3–5, 5–7, 7-9, 9-11, and 11-13 weeks) to further evaluate the association between treatment timing and survival.

A propensity score-matched analysis was then performed to match patients in earlier and later adjuvant chemotherapy groups using similar methods as those previously described.^15^ Briefly, propensity scores were developed, defined as the probability of treatment with early versus later adjuvant chemotherapy, conditional on the same covariates as in our multivariable Cox regression model with penalized smoothing splines. A radius-matching algorithm with a caliper of 0.01 was applied to identify the most appropriately matched pairs. After matching, balance was assessed using absolute standardized differences, and OS of the two groups was assessed using Kaplan-Meier analysis. Baseline characteristics were evaluated using Student's *t* test or Wilcoxon rank sum test for continuous variables and Pearson's χ^2^ test or Fisher's exact test for categorical variables.

A sensitivity analysis was performed by restricting the cohort to patients who were discharged within 4 days of surgery to account for postoperative recovery time influencing the association between timing of adjuvant chemotherapy. This analysis was conducted using multivariable Cox proportional hazards models and propensity score–matched analysis.

A subgroup analysis was conducted among patients with available data on minimally invasive surgical approaches (limited to cases from 2010 onward) using video-assisted thoracoscopic surgery and robotic approaches. This analysis was conducted using multivariable Cox proportional hazards models and propensity score–matched analysis.

All statistical analyses were performed using Stata/MP software, version 17.0 for Mac (StataCorp, College Station, TX). A two-sided *P* value of 0.05 was used to define significance.

## Results

**Figure 1:**
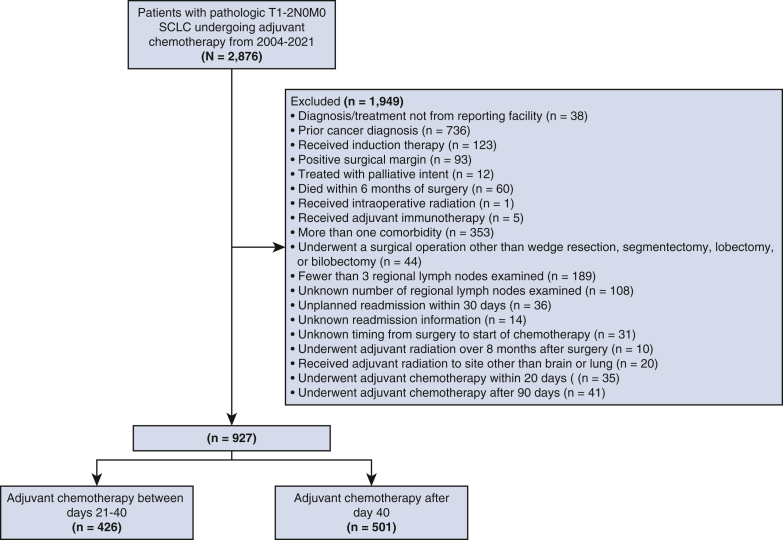
Flow diagram showing schema of study patient selection. *SCLC*, Small cell lung cancer.

Between 2004 and 2021, 2876 patients underwent surgical resection for pathologic stage T1-2N0M0 SCLC. Among these, 927 patients (32.2%) met study inclusion criteria (Figure 1). Median time to adjuvant chemotherapy was 41 days (interquartile range [IQR], 34, 53). 426 patients (46.0%) received “earlier” (time to adjuvant chemotherapy 21-40 days after surgery) adjuvant chemotherapy and 501 patients (54.0%) received “later” (time to adjuvant chemotherapy 41 or more days after surgery) adjuvant chemotherapy.

Table 1 details the baseline and perioperative characteristics of the patient cohort stratified by whether adjuvant chemotherapy was initiated before or after 41 days post-surgery. Table E1 depicts the predictors of later (time to adjuvant chemotherapy 41 or more days after surgery) adjuvant chemotherapy. Table E2 shows the surgical characteristics of the entire cohort.

**Table 1:** Baseline and demographic characteristics for patients who underwent complete resection for pT1-2N0M0 SCLC, stratified by earlier versus later adjuvant chemotherapy

Characteristic	Earlier adjuvant chemotherapy (n = 426)	Later adjuvant chemotherapy (n = 501)	*P*
Age (y), median (IQR)	66.0 (60.0, 71.0)	66.0 (60.0, 72.0)	.26
Sex, No. (%)			.36
Male	187 (43.9)	205 (40.9)	
Female	239 (56.1)	296 (59.1)	
Race, No. (%)			.57
White	397 (93.2)	460 (91.8)	
Black	16 (3.8)	26 (5.2)	
Other	12 (2.8)	13 (2.6)	
Unknown	<10	<10	
Year of diagnosis, median (IQR)	2016 (2012, 2019)	2017.0 (2013, 2019)	.01
Insurance Type, No. (%)			.92
Private	129 (30.3)	157 (31.3)	
Government	290 (68.1)	157 (66.7)	
Not Insured	<10	<10	
Unknown	<10	<10	
Facility type, No. (%)			.66
Community Cancer Program	25 (5.9)	24 (4.8)	
Comprehensive Community Cancer Program	160 (37.6)	195 (38.9)	
Academic/Research Program	149 (35.0)	186 (37.1)	
Integrated Network Cancer Program	92 (21.6)	96 (19.2)	
Distance from facility (miles), median (IQR)	10.9 (5.6, 22.6)	12.1 (5.9, 27.6)	.25
CDCC score, No. (%)			.36
0	232 (54.5)	288 (57.5)	
1	194 (45.5)	213 (42.5)	
Tumor Grade, No. (%)			.73
Well Differentiated	<10	<10	
Moderately Differentiated	<10	11 (2.2)	
Poorly Differentiated	155 (36.4)	181 (36.1)	
Undifferentiated	102 (23.9)	120 (24.0)	
Unknown	161 (37.8)	188 (37.5)	
Tumor Size (cm), median (IQR)	20.0 (15.0, 28.0)	21.0 (15.0, 30.0)	.18
Tumor Location, No. (%)			.90
Main Bronchus	<10	<10	
Right Upper Lobe	153 (35.9)	185 (36.9)	
Right Middle Lobe	26 (6.1)	35 (7.0)	
Right Lower Lobe	66 (15.5)	70 (14.0)	
Left Upper Lobe	109 (25.6)	135 (26.9)	
Left Lower Lobe	61 (14.3)	69 (13.8)	
Overlapping Lesion	<10	<10	
Unknown	<10	<10	
Surgery Type, No. (%)			.13
Wedge Resection	65 (15.3)	59 (11.8)	
Segmental Resection	18 (4.2)	14 (2.8)	
Lobectomy	343 (80.5)	428 (85.4)	
Surgical Approach			.33
Open	116 (27.2)	157 (31.3)	
VATS	117 (27.5)	125 (25.0)	
Robot	83 (19.5)	89 (17.8)	
Unknown	110 (25.8)	130 (25.9)	
Timing to adjuvant chemotherapy (d), median (IQR)	33 (28-36)	51 (46-61)	<.001
Pathologic T Stage, No. (%)			.66
T1a	40 (9.4)	46 (9.2)	
T1b	163 (38.3)	169 (33.7)	
T1c	97 (22.8)	121 (24.2)	
T2a	98 (23.0)	126 (25.1)	
T2b	28 (6.6)	39 (7.8)	
Adjuvant Radiation, No. (%)			.13
No Radiation	315 (73.9)	390 (77.8)	
PCI	101 (23.7)	93 (18.6)	
Lung	10 (2.3)	17 (3.4)	
PCI and Lung	<10	<10	
Median Income			.80
<$46,227	66 (15.5)	72 (14.4)	
$46,277 - $57,856	92 (21.6)	98 (19.6)	
$57,857 - $74,062	85 (20.0)	108 (21.6)	
≥$74,063	115 (27.0)	140 (27.9)	
Unknown	68 (16.0)	83 (16.6)	
Education, % without HS diploma			.30
>15.3%	68 (16.0)	77 (15.4)	
9.1% - 15.2%	93 (21.8)	123 (24.6)	
5.0% – 9.0%	108 (25.4)	136 (27.1)	
<5.0%	91 (21.4)	84 (16.8)	
Unknown	66 (15.5)	81 (16.2)	

*CDCC*, Charlson/Deyo comorbidity condition; *PCI*, Prophylactic cranial irradiation; *VATS*, Video-assisted thoracic surgery.

**Figure 2:**
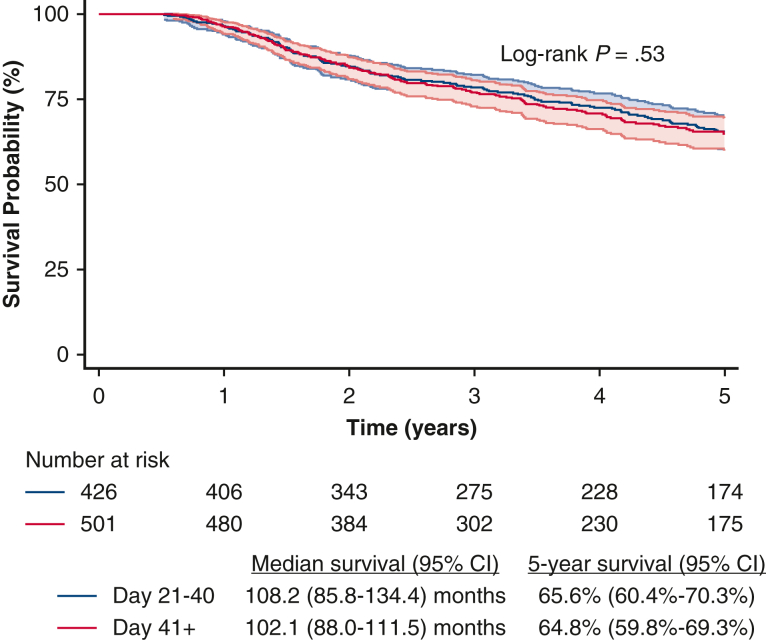
Overall survival of patients with pT1-2N0M0 small cell lung cancer, stratified by earlier versus later adjuvant chemotherapy. CI = 95%.

**Figure 3:**
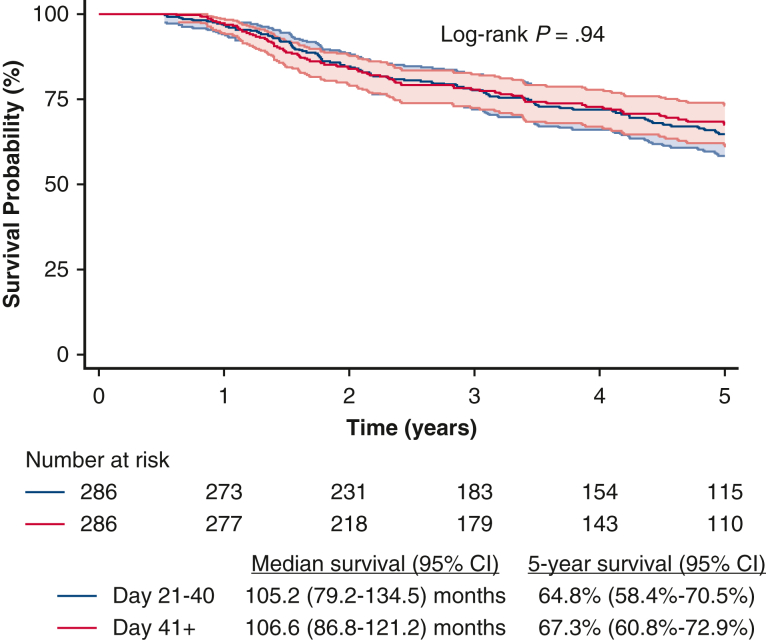
Overall survival of patients with pT1-2N0M0 small cell lung cancer, stratified by earlier versus later adjuvant chemotherapy: propensity score-matched analysis. CI = 95%.

Unadjusted Kaplan-Meier analysis demonstrated that there were no significant differences in median and 5-year OS between patients who received earlier adjuvant chemotherapy and those who received later adjuvant chemotherapy (Figure 2). In multivariable analysis, there was no significant difference in survival between the earlier and later adjuvant chemotherapy groups (Table 2). Similar findings were seen when adjuvant chemotherapy timing was modeled as a categorical variable in 2-week intervals (Table E3). Propensity score matching was used to create two groups of 286 patients each who underwent earlier or later adjuvant chemotherapy. The two groups were well matched with regard to baseline characteristics, and all absolute standardized differences were less than or equal to 13.5 (Table 3). There were no significant differences in baseline characteristics. There were no significant differences in median and 5-year OS between the earlier and later adjuvant chemotherapy groups (Figure 3).

**Table 2:** Independent predictors of overall survival after Cox proportional hazards adjustment for patients who have undergone complete resection for pT1-2N0M0 SCLC with adjuvant chemotherapy

Characteristic	Hazard ratio	95% CI	*P*
Age (y)	1.04	1.02, 1.06	.00
Female *v* male	0.91	0.71, 1.15	.43
Race (ref = white)			
Black	0.72	0.36, 1.44	.35
Other	0.77	0.34, 1.77	.54
Year of diagnosis (per y)	0.99	0.97, 1.02	.66
Insurance Type (ref = private)			
Government	1.32	0.98, 1.76	.07
Not Insured	5.32	2.07, 13.67	.00
Facility type (ref = community cancer program)			
Comprehensive Community Cancer Program	0.93	0.57, 1.51	.75
Academic/Research Program	0.85	0.51, 1.40	.52
Integrated Network Cancer Program	0.92	0.54, 1.55	.76
Distance from facility (per mile)	1.00	1.00, 1.01	.02
CDCC score (ref = 0)			
1	1.06	0.84, 1.34	.60
Tumor Size (per cm)	1.00	0.98, 1.02	.90
Tumor Location (ref = right middle lobe)			
Right Upper Lobe	0.57	0.36, 0.91	.02
Right Lower Lobe	0.98	0.59, 1.62	.94
Left Upper Lobe	0.71	0.44, 1.14	.16
Left Lower Lobe	0.78	0.46, 1.31	.35
Overlapping Lesion of the Lung	1.19	0.26, 5.41	.83
Surgery Type (ref = lobectomy)			
Wedge Resection	1.27	0.91, 1.76	.16
Segmental Resection	0.91	0.43, 1.90	.80
Pathologic T Stage (ref = T1a)			
T1b	1.02	0.64, 1.61	.94
T1c	1.05	0.59, 1.87	.87
T2a	1.53	0.83, 2.80	.17
T2b	1.77	0.68, 4.59	.24
Adjuvant Radiation (ref = no radiation)			
PCI	1.06	0.81, 1.39	.65
Lung	1.36	0.74, 2.49	.32
Median Income (ref = <$46,227)			
$46,277 - $57,856	1.00	0.71, 1.42	1.00
$57,857 - $74,062	0.73	0.50, 1.08	.11
≥$74,063	0.80	0.53, 1.22	.30
Education, % without HS diploma (ref = >15.3%)			
9.1% - 15.2%	1.07	0.74, 1.54	.73
5.0% – 9.0%	1.47	1.01, 2.15	.05
<5.0%	0.99	0.62, 1.57	.96
Later Adjuvant Chemotherapy (ref = earlier)	1.06	0.84, 1.33	.62

*HR*, Hazards ratio; *95% CI*, 95% confidence interval; *CDCC*, Charlson/Deyo comorbidity condition; *PCI*, Prophylactic cranial irradiation.

**Table 3:** Propensity-matched preoperative and demographic characteristics for patients who underwent complete resection for pT1-2N0M0 SCLC, stratified by earlier versus later adjuvant chemotherapy

Characteristic	Earlier adjuvant chemotherapy (n = 286)	Later adjuvant chemotherapy (n = 286)	Absolute standardized difference (%)	*P*
Age (y), median (IQR)	66.0 (60.0, 71.0)	66.0 (60.0, 71.0)	1.8	.73
Sex, No. (%)				.61
Male	119 (41.6)	125 (43.7)	4.2	
Female	167 (58.4)	161 (56.3)	4.2	
Race, No. (%)				.95
White	264 (92.3)	264 (92.3)	0.0	
Black	14 (4.9)	15 (5.2)	1.7	
Other	<10	<10	2.2	
Year of diagnosis, median (IQR)	2016.0 (2012.0, 2019.0)	2016.0 (2012.0, 2019.0)	4.2	.37
Insurance Type, No. (%)				.96
Private	91 (31.8)	88 (30.8)	2.3	
Government	193 (67.5)	196 (68.5)	2.2	
Not Insured	<10	<10	0.0	
Facility type, No. (%)				.79
Community Cancer Program	14 (4.9)	19 (6.6)	7.8	
Comprehensive Community Cancer Program	114 (39.9)	114 (39.9)	0.0	
Academic/Research Program	105 (36.7)	98 (34.3)	5.1	
Integrated Network Cancer Program	53 (18.5)	55 (19.2)	1.7	
Distance from facility (miles), mean (SD)	10.9 (6.0, 22.8)	11.6 (5.4, 26.1)	4.7	.81
CDCC score, No. (%)				.45
0	169 (59.1)	160 (55.9)	6.3	
1	117 (40.9)	126 (44.1)	6.3	
Tumor Grade, No. (%)				.95
Well Differentiated	<10	<10	0.0	
Moderately Differentiated	<10	<10	5.3	
Poorly Differentiated	108 (37.8)	105 (36.7)	2.2	
Undifferentiated	71 (24.8)	71 (24.8)	0.0	
Unknown	102 (35.7)	103 (36.0)	0.7	
Tumor Size (cm), median (IQR)	20.0 (15.0, 28.0)	20.0 (15.0, 28.0)	0.0	.99
Tumor Location, No. (%)				.94
Right Upper Lobe	105 (36.7)	102 (35.7)	2.2	
Right Middle Lobe	19 (6.6)	18 (6.3)	1.4	
Right Lower Lobe	42 (14.7)	49 (17.1)	6.9	
Left Upper Lobe	80 (28.0)	75 (26.2)	4.0	
Left Lower Lobe	40 (14.0)	41 (14.3)	1.0	
Overlapping Lesion of the Lung	<10	<10	6.1	
Surgery Type, No. (%)				.96
Wedge Resection	39 (13.6)	40 (14.0)	1.0	
Segmental Resection	<10	<10	1.9	
Lobectomy	240 (83.9)	238 (83.2)	1.9	
Surgical Approach				.12
Open	79 (27.6)	96 (33.6)	13.1	
VATS	81 (28.3)	64 (22.4)	13.5	
Robot	52 (18.2)	44 (15.4)	7.2	
Unknown	74 (25.9)	82 (28.7)	6.4	
Timing to adjuvant chemotherapy (d), median (IQR)	33 (28, 36)	52 (46, 62)	N/A	<.001
Pathologic T Stage, No. (%)				.99
T1a	22 (7.7)	24 (8.4)	2.4	
T1b	108 (37.8)	107 (37.4)	0.7	
T1c	71 (24.8)	71 (24.8)	0.0	
T2a	67 (23.4)	64 (22.4)	2.5	
T2b	18 (6.3)	20 (7.0)	2.7	
Adjuvant Radiation, No. (%)				1.00
No Radiation	214 (74.8)	213 (74.5)	0.8	
PCI	64 (22.4)	65 (22.7)	0.9	
Lung	<10	<10	0.0	
Median Income				.77
< $46,227	51 (17.8)	60 (21.0)	8.8	
$46,277 - $57,856	69 (24.1)	71 (24.8)	1.6	
$57,857 - $74,062	72 (25.2)	67 (23.4)	4.0	
≥74,063	94 (32.9)	88 (30.8)	4.5	
Education, % without HS diploma				.93
>15.3%	54 (18.9)	58 (20.3)	3.8	
9.1% - 15.2%	78 (27.3)	82 (28.7)	3.1	
5.0% – 9.0%	88 (30.8)	84 (29.4)	3.0	
<5.0%	66 (23.1)	62 (21.7)	3.3	

*IQR*, Interquartile range; *CDCC*, charlson/Deyo comorbidity condition; *PCI*, Prophylactic cranial irradiation.

**Figure E2:**
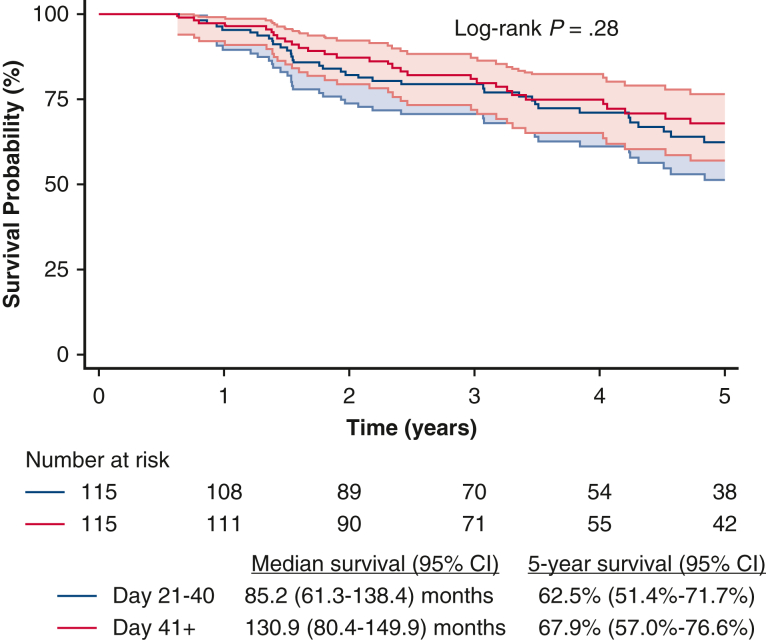
Overall survival of patients with pT1-2N0M0 small cell lung cancer who were discharged within 4 days of surgery, stratified by earlier versus later adjuvant chemotherapy: propensity score-matched analysis. CI = 95%.

In a sensitivity analysis limited to patients discharged within 4 days from surgery, multivariable analysis showed no significant difference in survival between the earlier and later adjuvant chemotherapy groups (Table E4) and when timing was analyzed in 2-week intervals (Table E5). Similar results were seen in propensity score-matched analysis (Figure E2).

**Figure E3:**
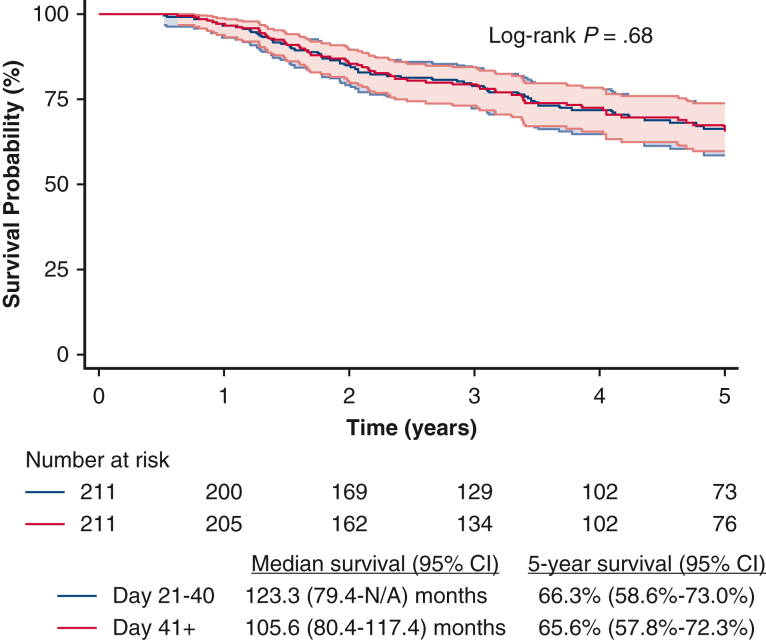
Overall survival of patients with pT1-2N0M0 small cell lung cancer with information available on surgical approach, stratified by earlier versus later adjuvant chemotherapy: propensity score-matched analysis. CI = 95%.

In a subgroup analysis limited to patients with known surgical approach (eg, open, video-assisted thoracoscopic surgery, and robotic), multivariable analysis showed no significant difference in survival between the earlier and later adjuvant chemotherapy groups (Table E6) and when timing was analyzed in 2-week intervals (Table E7). Similar results were seen in propensity score-matched analysis (Figure E3).

**Figure 4:**
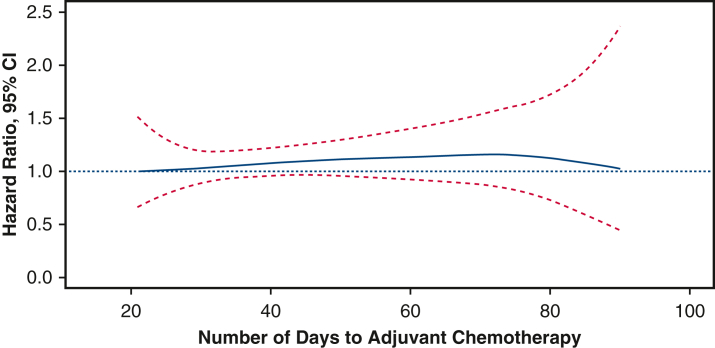
Multivariable Cox regression analysis with a penalized smoothing spline function for time elapsed from surgery to adjuvant chemotherapy for patients with pT1-2N0M0 SCLC.

Next, we used Cox proportional hazards regression to examine the implications of later adjuvant chemotherapy for all-cause mortality. We used penalized smoothing splines to capture nonlinearities in the relationship between days elapsed from surgery to adjuvant chemotherapy and mortality. For this analysis, we used individuals who underwent adjuvant chemotherapy at 21 days as the reference group. There was no significant association observed between time to adjuvant chemotherapy and overall survival across the evaluated time range (Figure 4). This analysis was performed in patients with one or less major co-morbidity.

## Discussion

In this study, we examined the relationship between the timing of adjuvant chemotherapy following complete resection and overall survival for early-stage node-negative SCLC. In this national analysis, patients whose adjuvant chemotherapy was given 41-90 days after surgery were found to have similar survival to those whose adjuvant chemotherapy was given 21-40 days after surgery in unadjusted, multivariable and propensity score-matched analyses. When we characterized the nonlinear relationship between timing of adjuvant chemotherapy and survival using multivariable Cox regression analysis with penalized smoothing splines, we found a similar hazard of death regardless of the time elapsed between surgery and initiation of adjuvant chemotherapy.

To our knowledge, the present study is the first to evaluate the impact of timing of adjuvant chemotherapy for patients who underwent complete resection (R0) for pathologic stage I-IIA (T1-2N0M0) SCLC. There have been previous studies that have investigated the optimal timing of adjuvant chemotherapy in (NSCLC); these studies found that delayed adjuvant chemotherapy was not associated with worse survival.^5^^,^^12^^,^^16^, ^17^, ^18^ When taken together with the literature in non–small cell lung cancer, the findings from the present study suggest that delayed administration of adjuvant chemotherapy for resected early-stage SCLC does not adversely affect survival. The study findings can be used to help with treatment decision making in the real world (eg, patients with a complicated postoperative course who may need more time to recover prior to receiving adjuvant chemotherapy) and also with future clinical trial design. Current study designs evaluating adjuvant therapies often only allow for a narrow timeframe for the administration of adjuvant chemotherapy.^19^ The study findings suggest that a broader range of time of administration of adjuvant chemotherapy can still be considered appropriate for carefully selected patients. There will also be further opportunities to evaluate the impact of timing of adjuvant therapy when Alliance Foundation Trial 61 (Adjuvant Chemotherapy and Immunotherapy for Completely Resected Small Cell Lung Cancer) launches.

The present study has the following strengths. By using the NCDB, we were able to create a larger national cohort that limited confounding by not including a heterogeneous group of stages and restricting our analysis to patients with no more than one major comorbidity. Furthermore, by using multivariable Cox regression analysis with penalized smoothing splines, we were able to characterize the nonlinear relationship between the timing of adjuvant chemotherapy and overall survival.

There are several limitations to the present study. First, as a retrospective analysis, there is the possibility of confounding variables that were not taken into account. Specifically, the NCDB does not have data on performance status, smoking history, pulmonary function, postoperative complications, or surgeon experience. We were unable to fully account for selection bias. As such, we are not able to evaluate whether patients with worse prognostic factors or with surgical complications were more often offered chemotherapy at a later timepoint. We did attempt to minimize this bias by including CDCC comorbidity score as a covariate in the multivariable and propensity-matched analysis, restricting the analysis to patients with no more than one comorbidity, restricting the analysis to patients who did not have any 30-day readmissions and performing a sensitivity analysis limited to patients who were discharged within 4 days from their surgery. Second, the NCDB does not have specific information on complications such as those related to wound healing. Prior literature suggests that preoperative and early administration of adjuvant chemotherapy is not associated with a significantly increased risk of wound healing problems.^20^, ^21^, ^22^, ^23^, ^24^ Third, the NCDB does not include data on the specific type and number of courses of chemotherapy administered. Fourth, the NCDB does not contain data on local and distant recurrence, disease-free survival, and cancer-specific survival. Finally, The NCDB does not contain information on chemotherapy doses, the specific chemotherapy agents or classes used, or the toxicities associated with treatment.

## Conclusions

In conclusion, in this national analysis, longer intervals between surgery of pT1-2N0M0 SCLC and adjuvant chemotherapy were not associated with worse survival.

## Conflict of Interest Statement

Dr Sands reports personal fees from AstraZeneca, Blueprint Medicines, Medtronic, Daiichi-Sankyo, Arcus, Jazz Pharmaceuticals, Pharmamar, Curadev, Takeda, and Boehringer Ingelhemi outside the submitted work. Dr Lanuti reports personal fees from AstraZeneca outside the submitted work. Dr Meador reports grants from Novartis and AstraZeneca outside the submitted work. Dr Yang is on the advisory boards for Genentech and AstraZeneca and received honorarium from AstraZeneca for speaking. Mr Mansur, Ms. Lee, and Ms. Potter reported no conflicts of interest.

The *Journal* policy requires editors and reviewers to disclose conflicts of interest and to decline handling or reviewing manuscripts for which they may have a conflict of interest. The editors and reviewers of this article have no conflicts of interest.

## Figures and Tables

**Table E1:** Independent predictors of later adjuvant chemotherapy for patients who underwent complete resection for pT1-2N0M0 SCLC

Characteristic	aOR	95% CI	*P*
Age (y)	1.02	0.99, 1.04	.16
Female *v* male	1.15	0.85, 1.57	.37
Race (ref = white)			
Black	1.19	0.58, 2.43	.64
Other	0.70	0.27, 1.81	.46
Year of diagnosis (per y)	1.04	1.01, 1.07	.02
Insurance Type (ref = private)			
Government	0.85	0.59, 1.22	.38
Not Insured	1.18	0.24, 5.78	.84
Facility type (ref = community cancer program)			
Comprehensive Community Cancer Program	1.05	0.55, 2.01	.89
Academic/Research Program	1.12	0.57, 2.17	.75
Integrated Network Cancer Program	0.97	0.48, 1.97	.94
Distance from facility (per mile)	1.00	1.00, 1.01	.27
CDCC score (ref = 0)			
1	0.90	0.67, 1.22	.51
Tumor Size (per cm)	1.00	0.97, 1.03	.80
Tumor Location (ref = right middle lobe)			
Right Upper Lobe	1.12	0.59, 2.12	.73
Right Lower Lobe	0.93	0.46, 1.88	.84
Left Upper Lobe	1.19	0.62, 2.29	.61
Left Lower Lobe	1.12	0.55, 2.29	.76
Overlapping Lesion of the Lung	1.01	0.05, 18.70	.99
Surgery Type (ref = lobectomy)			
Wedge Resection	0.67	0.43, 1.05	.08
Segmental Resection	0.41	0.17, 1.00	.05
Pathologic T Stage (ref = T1a)			
T1b	1.14	0.64, 2.02	.67
T1c	1.31	0.62, 2.78	.48
T2a	1.45	0.64, 3.27	.37
T2b	1.53	0.40, 5.76	.53
Adjuvant Radiation (ref = no radiation)			
PCI	0.78	0.54, 1.13	.19
Lung	1.21	0.49, 2.98	.67
Median Income (ref = <46,227)			
$46,277 - $57,856	1.04	0.65, 1.68	.86
$57,857 - $74,062	1.41	0.84, 2.36	.19
≥$74,063	1.77	1.00, 3.13	.05
Education, % without HS diploma (ref = >15.3%)			
9.1% - 15.2%	1.00	0.62, 1.60	.99
5.0% – 9.0%	0.81	0.49, 1.35	.42
<5.0%	0.47	0.25, 0.87	.02

*aOR*, Adjusted odds ratio; *95% CI*, 95% confidence interval; *CDCC,* Charlson/Deyo comorbidity condition; *PCI*, Prophylactic cranial irradiation.

**Table E2:** Surgical characteristics for patients with pT1-2N0M0 SCLC who underwent adjuvant chemotherapy, stratified by surgery type

Characteristic	Wedge resection n = 124	Segmentectomy n = 32	Lobectomy n = 771	*P*
Tumor Size (cm), median (IQR)	1.6 (1.2-2.1)	2.0 (1.3, 2.4)	2.1 (1.5-3.0)	<.001
Lymph Nodes Evaluated, median (IQR)	6 (4-10)	8 (4.5-11.5)	10 (6-16)	<.001
Tumor Location, No. (%)				.58
Main Bronchus	<10	<10	<10	
Right Upper Lobe	45 (36.29)	<10	285 (37.0)	
Right Middle Lobe	<10	<10	54 (7.0)	
Right Lower Lobe	20 (16.1)	<10	108 (14.0)	
Left Upper Lobe	32 (25.8)	<10	204 (26.5)	
Left Lower Lobe	20 (16.1)	<10	103 (13.4)	
Overlapping lesion	<10	<10	<10	
Unknown	<10	<10	13 (1.7)	
Timing to adjuvant chemotherapy (d), median (IQR)	40 (33-48.5)	40 (35.5-51.5)	42 (34-53)	.28
Pathologic T Stage, No. (%)				.001
T1a	19 (15.3)	<10	62 (8.0)	
T1b	52 (41.9)	10 (31.3)	270 (35.0)	
T1c	15 (12.1)	<10	194 (25.2)	
T2a	36 (29.0)	<10	183 (23.7)	
T2b	<10	<10	62 (8.0)	
Minimally Invasive Surgery, No (%)				.46
Open	38 (30.7)	<10	227 (29.4)	
VATS	39 (31.5)	<10	196 (25.4)	
Robot	24 (19.4)	10	138 (17.9)	
Unknown	23 (18.6)	<10	210 (27.2)	

**Table E3:** Independent predictors of overall survival after Cox proportional hazards adjustment for patients who have undergone complete resection for pT1-2N0M0 SCLC with adjuvant chemotherapy stratified by 2-week intervals

Characteristic	Hazard ratio	95% CI	*P*
Age (y)	1.04	1.02, 1.06	.00
Female *v* male	0.91	0.72, 1.16	.45
Race (ref = white)			
Black	0.71	0.35, 1.43	.34
Other	0.78	0.34, 1.78	.55
Year of diagnosis (per y)	0.99	0.96, 1.02	.64
Insurance Type (ref = private)			
Government	1.31	0.98, 1.76	.07
Not insured	5.42	2.09, 14.06	.00
Facility type (ref = community cancer program)			
Comprehensive Community Cancer Program	0.91	0.56, 1.48	.70
Academic/Research Program	0.84	0.51, 1.39	.50
Integrated Network Cancer Program	0.90	0.53, 1.53	.70
Distance from facility (per mile)	1.00	1.00, 1.01	.02
CDCC score (ref = 0)			
1	1.06	0.84, 1.33	.64
Tumor Size (per cm)	1.00	0.98, 1.02	.87
Tumor Location (ref = right middle lobe)			
Right Upper Lobe	0.57	0.36, 0.91	.02
Right Lower Lobe	0.99	0.60, 1.63	.96
Left Upper Lobe	0.71	0.44, 1.14	.16
Left Lower Lobe	0.77	0.46, 1.30	.33
Overlapping Lesion of the Lung	1.23	0.27, 5.68	.79
Surgery Type (ref = lobectomy)			
Wedge Resection	1.25	0.90, 1.75	.18
Segmental Resection	0.87	0.42, 1.83	.72
Pathologic T Stage (ref = T1a)			
T1b	1.01	0.63, 1.60	.98
T1c	1.02	0.57, 1.83	.93
T2a	1.49	0.81, 2.74	.20
T2b	1.75	0.67, 4.58	.25
Adjuvant Radiation (ref = No radiation)			
PCI	1.05	0.80, 1.37	.74
Lung	1.34	0.73, 2.47	.35
Median Income (ref = <$46,227)			
$46,277 - $57,856	1.00	0.71, 1.42	.99
$57,857 - $74,062	0.74	0.50, 1.08	.12
≥$74,063	0.78	0.51, 1.19	.25
Education, % without HS diploma (ref = >15.3%)			
9.1% - 15.2%	1.06	0.74, 1.53	.75
5.0% – 9.0%	1.49	1.02, 2.17	.04
<5.0%	1.01	0.64, 1.62	.95
Adjuvant Chemotherapy (ref = 3-5 wk)			
5-7 wk	1.13	0.85, 1.49	.41
7-9 wk	1.07	0.76, 1.50	.71
9-11 wk	1.36	0.88, 2.11	.17
11-13 wk	0.99	0.49, 1.99	.97

*95% CI*, 95% Confidence interval; *CDCC*, Charlson/Deyo comorbidity condition; *PCI*, Prophylactic cranial irradiation.

**Table E4:** Independent predictors of overall survival after Cox proportional hazards adjustment for patients who have undergone complete resection for pT1-2N0M0 SCLC with adjuvant chemotherapy and were discharged within 4 day of surgery

Characteristic	Hazards ratio	95% CI	*P*
Age (y)	1.04	1.01, 1.06	.01
Female *v* male	1.03	0.72, 1.47	.88
Race (ref = white)			
Black	0.58	0.18, 1.89	.36
Other	0.76	0.10, 5.62	.79
Year of diagnosis (per y)	0.99	0.94, 1.03	.61
Insurance Type (ref = private)			
1	1.06	0.68, 1.64	.80
2	3.33	0.42, 26.57	.26
Facility type (ref = community cancer program)			
Comprehensive Community Cancer Program	0.88	0.36, 2.14	.78
Academic/Research Program	0.77	0.31, 1.89	.57
Integrated Network Cancer Program	0.94	0.37, 2.38	.90
Distance from facility (per mile)	1.01	1.00, 1.01	.00
CDCC score (ref = 0)			
1	1.08	0.77, 1.53	.66
Tumor Size (per cm)	1.03	0.99, 1.06	.11
Tumor Location (ref = right middle lobe)			
Right Upper Lobe	0.53	0.28, 1.01	.06
Right Lower Lobe	0.70	0.35, 1.41	.32
Left Upper Lobe	0.58	0.30, 1.15	.12
Left Lower Lobe	0.62	0.30, 1.28	.20
Surgery Type (ref = lobectomy)			
Wedge Resection	1.50	0.98, 2.31	.06
Segmental Resection	1.12	0.44, 2.81	.82
Pathologic T Stage (ref = T1a)			
T1b	0.82	0.42, 1.58	.55
T1c	0.76	0.33, 1.76	.53
T2a	1.15	0.49, 2.72	.74
T2b	0.88	0.22, 3.59	.86
Adjuvant Radiation (ref = no radiation)			
PCI	0.89	0.58, 1.38	.61
Lung	1.08	0.40, 2.93	.88
Later Adjuvant Chemotherapy (ref = earlier)	0.89	0.62, 1.27	.52

*95% CI*, 95% Confidence interval; *CDCC*, Charlson/Deyo comorbidity condition; *PCI*, Prophylactic cranial irradiation.

**Table E5:** Independent predictors of overall survival after Cox proportional hazards adjustment for patients who have undergone complete resection for pT1-2N0M0 SCLC with adjuvant chemotherapy stratified by 2-week intervals and were discharged within 4 day of surgery

Characteristic	Hazards ratio	95% CI	*P*
Age (y)	1.03	1.01, 1.06	.01
Female *v* male	1.01	0.70, 1.45	.95
Race (ref = white)			
Black	0.54	0.16, 1.81	.32
Other	0.75	0.10, 5.58	.78
Year of diagnosis (per y)	0.99	0.94, 1.04	.60
Insurance Type (ref = private)			
Government	1.06	0.68, 1.65	.81
No Insurance	6.34	0.60, 66.73	.12
Facility type (ref = community cancer program)			
Comprehensive Community Cancer Program	0.86	0.36, 2.05	.73
Academic/Research Program	0.74	0.31, 1.80	.51
Integrated Network Cancer Program	0.92	0.37, 2.31	.86
Distance from facility (per mile)	1.01	1.00, 1.01	.00
CDCC score (ref = 0)			
1	1.07	0.76, 1.51	.70
Tumor Size (per cm)	1.03	0.99, 1.06	.12
Tumor Location (ref = right middle lobe)			
Right Upper Lobe	0.54	0.28, 1.03	.06
Right Lower Lobe	0.72	0.36, 1.45	.36
Left Upper Lobe	0.61	0.31, 1.20	.16
Left Lower Lobe	0.63	0.31, 1.30	.21
Surgery Type (ref = lobectomy)			
Wedge Resection	1.50	0.97, 2.31	.07
Segmental Resection	1.09	0.43, 2.76	.86
Pathologic T Stage (ref = T1a)			
T1b	0.80	0.41, 1.55	.51
T1c	0.74	0.32, 1.71	.48
T2a	1.11	0.47, 2.64	.81
T2b	0.87	0.21, 3.57	.85
Adjuvant Radiation (ref = no radiation)			
PCI	0.91	0.59, 1.41	.68
Lung	1.06	0.39, 2.89	.91
Adjuvant Chemotherapy (ref = 3-5 wk)			
5-7 wk	0.92	0.62, 1.37	.68
7-9 wk	0.98	0.57, 1.69	.95
9-11 wk	1.11	0.52, 2.40	.78
11-13 wk	0.39	0.08, 1.98	.26

*HR*, Hazards ratio; *95% CI*, 95% confidence interval; *CDCC*, Charlson/Deyo comorbidity condition; *PCI*, Prophylactic cranial irradiation.

**Table E6:** Independent predictors of overall survival after Cox proportional hazards adjustment for patients who have undergone complete resection for pT1-2N0M0 SCLC with adjuvant chemotherapy and had information on surgical approach

Characteristic	Hazards ratio	95% CI	*P*
Age (y)	1.04	1.02, 1.06	.00
Female *v* male	0.82	0.61, 1.11	.20
Race (ref = white)			
Black	0.56	0.22, 1.41	.22
Other	0.78	0.24, 2.55	.69
Year of diagnosis (per y)	1.03	0.98, 1.08	.32
Insurance Type (ref = private)			
Government	1.29	0.89, 1.86	.18
Not Insured	7.16	2.42, 21.21	.00
Facility type (ref = community cancer program)			
Comprehensive Community Cancer Program	0.98	0.46, 2.09	.96
Academic/Research Program	0.87	0.40, 1.86	.72
Integrated Network Cancer Program	0.89	0.40, 1.98	.78
Distance from facility (per mile)	1.00	1.00, 1.01	.04
CDCC score (ref = 0)			
1	0.93	0.70, 1.24	.64
Tumor Size (per cm)	0.99	0.97, 1.02	.65
Tumor Location (ref = right middle lobe)			
Right Upper Lobe	0.73	0.39, 1.37	.33
Right Lower Lobe	1.24	0.65, 2.37	.52
Left Upper Lobe	0.72	0.38, 1.37	.31
Left Lower Lobe	0.87	0.43, 1.73	.69
Overlapping Lesion of the Lung	1.79	0.36, 8.92	.48
Surgery Type (ref = lobectomy)			
Wedge Resection	1.16	0.78, 1.71	.47
Segmental Resection	0.41	0.13, 1.31	.13
Pathologic T Stage (ref = T1a)			
T1b	1.13	0.62, 2.04	.69
T1c	1.53	0.74, 3.14	.25
T2a	1.69	0.81, 3.54	.17
T2b	2.06	0.64, 6.59	.23
Adjuvant Radiation (ref = No radiation)			
PCI	1.36	0.96, 1.91	.08
Lung	1.34	0.61, 2.95	.47
Surgical Approach (ref = open)			
VATS	1.04	0.75, 1.45	.81
Robot	0.94	0.62, 1.44	.78
Later Adjuvant Chemotherapy (ref = earlier)	1.01	0.76, 1.34	.95

*HR*, Hazards ratio; *95% CI*, 95% confidence interval; *CDCC*, Charlson/Deyo comorbidity condition; *PCI,* Prophylactic cranial irradiation; *VATS*, video-assisted thoracic surgery.

**Table E7:** Independent predictors of overall survival after Cox proportional hazards adjustment for patients who have undergone complete resection for pT1-2N0M0 SCLC with adjuvant chemotherapy stratified by 2-week intervals and had information on surgical approach

Characteristic	Hazards ratio	95% CI	*P*
Age (y)	1.04	1.02, 1.06	.00
Female *v* male	0.81	0.60, 1.10	.18
Race (ref = white)			
Black	0.54	0.21, 1.36	.19
Other	0.78	0.24, 2.54	.68
Year of diagnosis (per y)	1.03	0.98, 1.08	.31
Insurance Type (ref = private)			
Government	1.32	0.91, 1.91	.15
Not Insured	7.04	2.31, 21.46	.00
Facility type (ref = community cancer program)			
Comprehensive Community Cancer Program	0.97	0.45, 2.08	.94
Academic/Research Program	0.86	0.40, 1.85	.70
Integrated Network Cancer Program	0.88	0.40, 1.96	.76
Distance from facility (per mile)	1.00	1.00, 1.01	.04
CDCC score (ref = 0)			
1	0.91	0.68, 1.21	.51
Tumor Size (per cm)	1.00	0.97, 1.02	.79
Tumor Location (ref = main bronchus)			
Right Upper Lobe	0.72	0.38, 1.36	.31
Right Lower Lobe	1.24	0.65, 2.37	.52
Left Upper Lobe	0.72	0.38, 1.38	.32
Left Lower Lobe	0.88	0.44, 1.77	.72
Overlapping Lesion of the Lung	1.95	0.39, 9.78	.42
Surgery Type (ref = lobectomy)			
Wedge Resection	1.12	0.76, 1.66	.56
Segmental Resection	0.39	0.12, 1.27	.12
Pathologic T Stage (ref = T1a)			
T1b	1.08	0.59, 1.97	.80
T1c	1.42	0.69, 2.94	.34
T2a	1.58	0.75, 3.34	.23
T2b	2.03	0.63, 6.55	.24
Adjuvant Radiation (ref = no radiation)			
PCI	1.34	0.95, 1.89	.10
Lung	1.37	0.62, 3.05	.44
Surgical Approach (ref = lobectomy)			
VATS	1.06	0.76, 1.47	.75
Robot	0.96	0.63, 1.46	.84
Adjuvant Chemotherapy (ref = 3-5 wk)			
5-7 wk	1.16	0.82, 1.64	.41
7-9 wk	0.84	0.54, 1.32	.46
9-11 wk	1.27	0.73, 2.23	.40
11-13 wk	1.16	0.56, 2.39	.70

*HR*, Hazards ratio; *95% CI*, 95% confidence interval; *CDCC*, Charlson/Deyo comorbidity condition; *PCI*, Prophylactic cranial irradiation; *VATS*, video-assisted thoracic surgery.
